# Structure and identification of the native PLP synthase complex from *Methanosarcina acetivorans* lysate

**DOI:** 10.1128/mbio.03090-24

**Published:** 2024-11-26

**Authors:** Angela Agnew, Ethan Humm, Kang Zhou, Robert P. Gunsalus, Z. Hong Zhou

**Affiliations:** 1 Department of Microbiology, Immunology & Molecular Genetics, University of California Los Angeles, Los Angeles, California, USA; 2 California NanoSystems Institute, University of California Los Angeles, Los Angeles, California, USA; 3 Department of Chemistry and Biochemistry, University of California Los Angeles, Los Angeles, California, USA; 4 UCLA-DOE Institute, Los Angeles, California, USA; University of Washington School of Medicine, Seattle, Washington, USA

**Keywords:** cryo-EM, cryoID, PLP synthase, *Methanosarcina acetivorans*

## Abstract

Many protein-protein interactions behave differently in biochemically purified forms as compared to their *in vivo* states. As such, determining native protein structures may elucidate structural states previously unknown for even well-characterized proteins. Here, we apply the bottom-up structural proteomics method, *cryoID*, toward a model methanogenic archaeon. While they are keystone organisms in the global carbon cycle and active members of the human microbiome, there is a general lack of characterization of methanogen enzyme structure and function. Through the *cryoID* approach, we successfully reconstructed and identified the native *Methanosarcina acetivorans* pyridoxal 5′-phosphate (PLP) synthase (PdxS) complex directly from cryogenic electron microscopy (cryo-EM) images of fractionated cellular lysate. We found that the native PdxS complex exists as a homo-dodecamer of PdxS subunits, and the previously proposed supracomplex containing both the synthase (PdxS) and glutaminase (PdxT) was not observed in cellular lysate. Our structure shows that the native PdxS monomer fashions a single 8α/8β TIM-barrel domain, surrounded by seven additional helices to mediate solvent and interface contacts. A density is present at the active site in the cryo-EM map and is interpreted as ribose 5-phosphate. In addition to being the first reconstruction of the PdxS enzyme from a heterogeneous cellular sample, our results reveal a departure from previously published archaeal PdxS crystal structures, lacking the 37-amino-acid insertion present in these prior cases. This study demonstrates the potential of applying the *cryoID* workflow to capture native structural states at atomic resolution for archaeal systems, for which traditional biochemical sample preparation is nontrivial.

**IMPORTANCE:**

Archaea are one of the three domains of life, classified as a phylogenetically distinct lineage. There is a paucity of known enzyme structures from organisms of this domain, and this is often exacerbated by characteristically difficult growth conditions and a lack of readily available molecular biology toolkits to study proteins in archaeal cells. As a result, there is a gap in knowledge concerning the mechanisms governing archaeal protein behavior and their impacts on both the environment and human health; case in point, the synthesis of the widely utilized cofactor pyridoxal 5′-phosphate (PLP; a vitamer of vitamin B6, which humans cannot produce). By leveraging the power of single-particle cryo-EM and map-to-primary sequence identification, we determine the native structure of PLP synthase from cellular lysate. Our workflow allows the (i) rapid examination of new or less characterized systems with minimal sample requirements and (ii) discovery of structural states inaccessible by recombinant expression.

## INTRODUCTION

While protein complexes from model eukaryotic and prokaryotic systems are highly represented in structural biology literature, there remain gaps in knowledge in cases of dynamic cellular processes and taxonomically neglected species. As members of one of the three domains of life alongside bacteria and eukaryotes, archaeal organisms (1) have been chronically understudied due to several issues that have hindered accurate documentation of the domain. These include, but are not limited to, differences in bacterial identification strategies relative to archaeal metabolism and cell structure, the frequent difficulty of culturing archaea, and the lack of a robust body of archaeal functional genomic annotations in existing databases (2). Furthermore, many of these species can be recalcitrant to traditional molecular biology techniques due to extremes of cell culture conditions (e.g., temperature, pH, osmolarity, and anaerobiosis) plus low cell yield. Recombinant approaches are not applicable when unique archaeal cofactors are required for protein function. While clonal and CRISPR-Cas9 techniques have been recently introduced for methanogenic archaeal clades, they are neither currently commercially available nor widely distributed across the range of archaeal model species (3–5). However, often these unique cases provide crucial evolutionary context for foundational biology.

Methanogens are a subset of the kingdom Euryarchaeota of the domain Archaea and are classified by their unique ability to anoxically produce methane. They are the primary source of biogenic methane on earth and drive the anoxic carbon cycle. These archaea are ubiquitous in anaerobic habitats, such as wetlands, marine sediments, sewage plants, and the GI tracts of cold- and warm-blooded animals (6). Methanogenesis is an ancient metabolism, with the first methanogen likely existing not long after bacteria and archaea diverged (7). Though still responsible for producing 1 gigaton of methane annually, much of the methane produced is subsequently metabolized by methanotrophic microorganisms living in adjoining anaerobic and aerobic environments (6). The activity of methanotrophs is still insufficient to offset combined biogenic and nonbiogenic sources of methane, such that the impact of microorganisms cannot be ignored in the effort to curtail greenhouse gas emissions.

Yet another feature of several methanogenic archaea is their relationship with the human microbiome. Archaeal species have now been identified from skin, the GI tract, and respiratory tract (8, 9). The consequence of cross-feeding with fermentative bacteria in this context is promoting the overgrowth of pathogenic microbes, yet this field of research is still in its infancy. Still, there are some methanogenic archaea now tied to periodontitis, irritable bowel syndrome, and colorectal cancer (10, 11). Since H_2_ is a methanogenic substrate, methanogens keep environmental concentrations of H_2_ low, energetically benefiting the fermentative metabolism of other bacteria. While these effects are not known to arise from virulence factors of the archaea themselves, these outcomes do result from their metabolic behavior and adherence to sites of infection. A mechanistic understanding of their key enzymes may give rise to therapeutic targets that can ameliorate these polymicrobial diseases.

**Fig 1:**
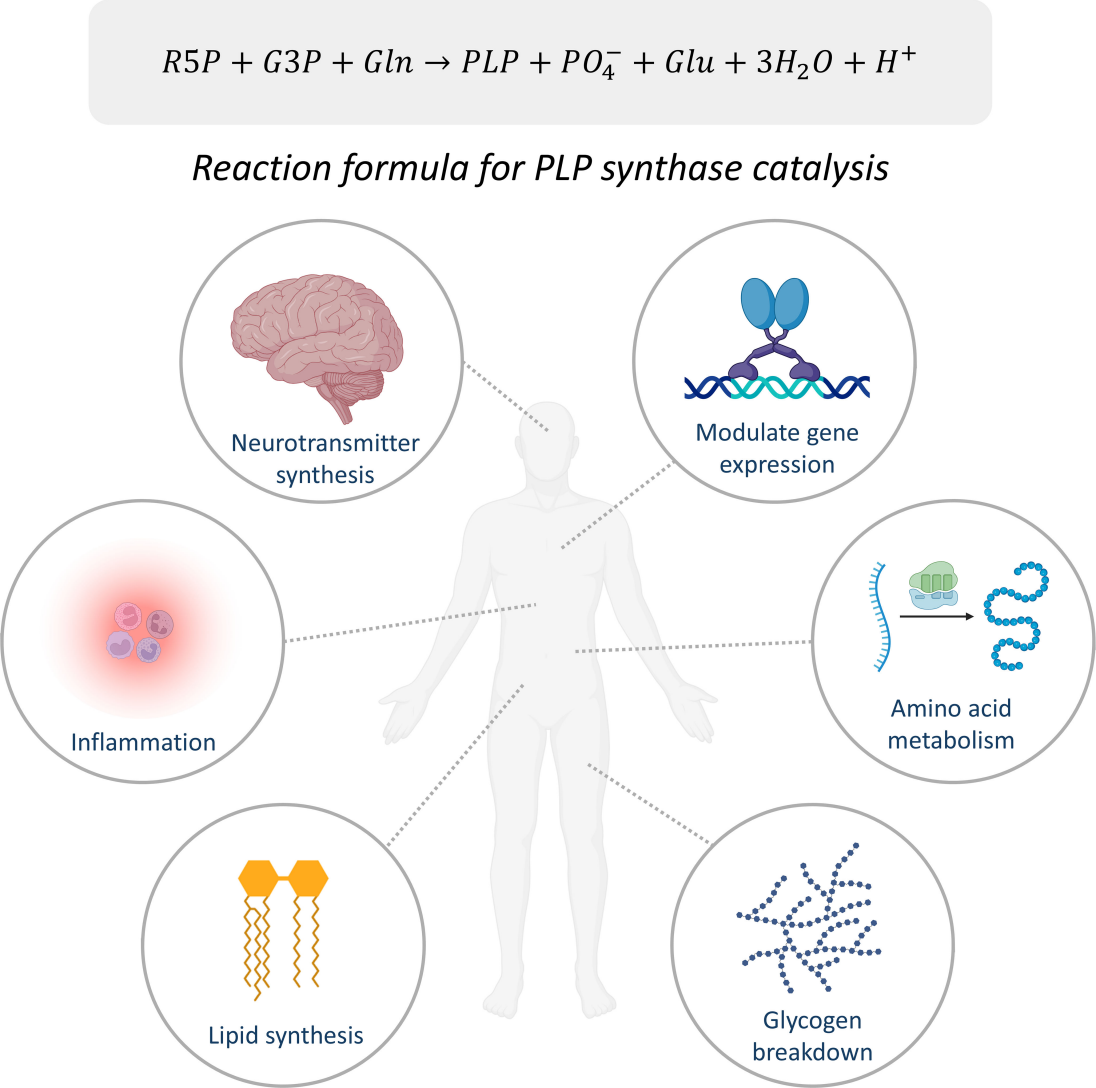
PLP is an essential cofactor for many basic cellular processes implicated in human health, including biosynthesis and metabolism of amino acids, carbohydrates, lipids, and nucleic acids; neurotransmitter production; modulating steroidal receptor gene expression; and regulating immune function and inflammatory responses (17–19).

One such target which we probe in this study is pyridoxal 5′-phosphate (PLP), a vitamer of vitamin B6, which is an essential cofactor for human neurological and immune health yet is one that humans and animals cannot natively synthesize. PLP is the biologically active state of vitamin B6, participating in over 160 crucial enzymatic processes, which include the metabolism of glycogen, amino acids, and lipids (Fig. 1) (12, 13). Particularly due to its roles in the production of neurotransmitters and modulation of interleukin-2, PLP deficiencies have been correlated with neurological disorders, such as epileptic encephalopathy, schizophrenia, and Parkinson’s disease, as well as immune system dysregulation and heightened inflammation (14, 15). Since humans can only obtain vitamin B6 from their diet or from endogenous microbial species, it is clear that microbiome composition and activity have an effect on vitamin B6 utilization (16). As components of the gut and oral microbiome, vitamin B6 production may represent another route through which archaea have specific relevance to human health outcomes. Furthermore, the fact that humans lack the genes for *de novo* vitamin B6 synthesis suggests that PLP synthases are a potentially druggable target for microbial disease. Some methanogenic archaea can grow mutualistically with environmental bacterial colonies, marking them for examination in the development of therapeutics against pathogenic bacterial species.

Of current structural biology techniques, cryogenic electron microscopy (cryo-EM) singularly enables us to achieve high-resolution structures of large-scale, frozen-hydrated protein complexes in their native states (20). Previous publications have pioneered a pipeline that allows for autonomous reconstruction and identification of unknown proteins from images of an enriched cellular milieu, using an in-house software termed *cryoID* (21–23). By avoiding extensive recombinant modifications, proteins may maintain the conformations, complexation, and chemical modifications crucial to their activity in living cells. To date, this method has not been applied to an archaeal system in pursuit of cryo-EM reconstruction of native proteins.

Here, we have applied the *cryoID* approach to study the model methanogen species *Methanosarcina acetivorans*, chosen for its comparatively rapid culture growth and established characterization, which is apt for exploratory studies. By directly imaging and identifying native cytosolic proteins, we have successfully determined the PdxS subunit of its pyridoxal 5′-phosphate (PLP) synthase. Our reconstruction of the dodecamer reveals departures from the only two known crystal structures from Euryarchaeota Archaea and demonstrates the promise of the *cryoID* workflow for samples from less characterized systems, particularly those from Archaea, where molecular biology approaches have been limited.

## RESULTS

### Cryo-EM reconstruction

**Fig 2:**
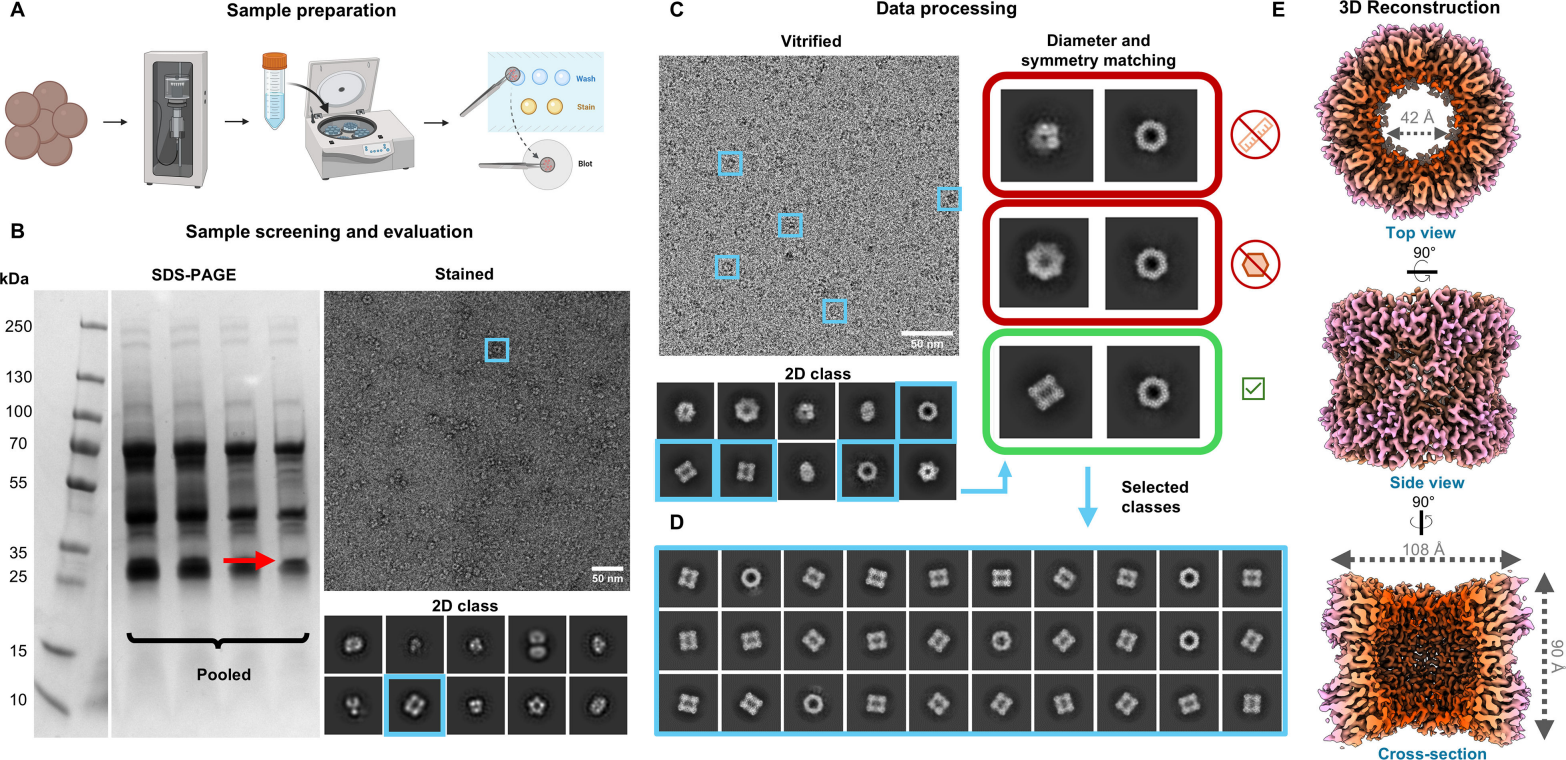
*CryoID* workflow for the reconstruction of PdxS. (**A**) *M. acetivorans* cells are lysed by sonication, fractionated by size, and applied to TEM grids. (**B**) Sample screening and evaluation. The left panel contains a Coomassie-stained SDS-PAGE gel with the selected fractions of interest, with the band corresponding to the PdxS molecular weight indicated by the red arrow. On the right, a representative negative stain TEM micrograph of this selected density gradient fraction and corresponding 2D class averages are displayed. An example PdxS particle and its corresponding 2D class are both boxed in blue. (**C–E**) Cryo data reconstruction workflow. An example cryo-EM micrograph (**C**) illustrates the extent of sample heterogeneity, with particles of interest corresponding to PdxS boxed in blue. Like in panel B, example PdxS particles and the corresponding 2D class are boxed in blue. These particles are also used in the panel “diameter and symmetry matching” for better illustration (**C–E**). Different 2D classes hypothesized to represent orthogonal views were compared based upon matching dimensions, symmetry compatibility, and ultimately reasonable *ab initio* reconstructions. In this example, the first two classes mismatch in their longest diameters, and the second two are a highly unlikely symmetry pair. (**D**) After selecting initial classes that represent top and side views, the data set was repicked with a Topaz model trained on the selected classes, giving rise to the second set of 2D classes exclusively showing different views of our chosen particle (**D**). Using D6 symmetric nonhomogenous reconstruction in cryoSPARC, we obtain the map seen in panel E. Density map is colored by cylindrical radius.

In efforts to simultaneously ensure a sufficiently high population of dominant species in this sample while preserving as many native interactions as possible, per the *cryoID* approach, a glycerol density gradient alone was used to fractionate the lysate of 100 µL of pelleted cells from a 50-mL culture. A 2-mL fraction window containing particles 8–12 nm in diameter was pooled for structural examination due to the promising 2D class averages noted in negative stain electron microscopy (EM) screening (Fig. 2B). Even after selecting a bespoke size range, resultant images of stained and frozen samples make evident the retained heterogeneity after separation (Fig. 2A through C). Similar classes were noted between negative stain and cryo-EM 2D averaging, and by employing a single-particle analysis approach with the cryoSPARC suite (24), we reconstructed a cryo-EM map to 3.38 Å with D6 symmetry (Fig. 2D and E). Even with just several views predominating, a high-resolution map could be reconstructed due to its high symmetry. This made it rather feasible to “mix and match” classes, which look extremely different from each other as putative top and side views, providing they have similar dimensions with high and compatible symmetries, and eventually discover an *ab initio* reconstruction that produced a rational and continuous density. After repicking the data set with a deep learning (Topaz) model trained on particles contributing to the best *ab initio* model, we successfully expanded our particle data set selected specifically for this bespoke species (25). Using the 3.38-Å resolution cryo-EM reconstruction, we could identify the particle as the 387-kD synthase (PdxS, UniProt accession no. Q8TQH6) subunit of the *M. acetivorans* pyridoxal 5′ phosphate synthase (previously shown to appear in supramolecular complex with its glutaminase subunit, PdxT [Table 1] [26, 27]) through the *cryoID* software (21) as well as model amino acids 11–301 of the monomeric subunit, thus bypassing the need for traditional mass spectrometry identification.

**TABLE 1:** Comparison of existing PLP structures and departing characteristics including acquisition methods and oligomeric states^*a*^

System	Domain	Structure method(s)	Recombinant expression	Glutaminase bound	37-aa insert present	Oligomeric state
*M. acetivorans*	A	cryo-EM	No	No	No	Homo 12-mer
*Arabidopsis thaliana*	E	XRD, cryo-EM	Yes	No	No	Homo 12-mer
*Saccharomyces cerevisiae*	E	XRD	Yes	No	No	Homo 6-mer
*Bacillus subtilis*	B	XRD	Yes	Yes	No	Hetero 24-mer
*Staphylococcus aureus*	B	XRD	Yes	Yes	No	Hetero 24-mer
*Plasmodium berghei*	E	XRD	Yes	Yes	No	Hetero 24-mer
*Thermotoga maritima*	B	XRD	Yes	Yes	No	Hetero 24-mer
*Thermus thermophilus*	B	XRD	Yes	No	No	Homo 12-mer
*Geobacillus kaustophilus*	B	XRD	Yes	No	No	Homo 12-mer
*Geobacillus stearothermophilus*	B	XRD	Yes	No	No	Homo 12-mer
*Mycobacterium tuberculosis*	B	XRD	Yes	No	No	Homo 12-mer
*Methanocaldococcus jannaschii*	A	XRD	Yes	No	Yes	Homo 12-mer
*Pyrococcus horikoshii*	A	XRD	Yes	No	Yes	Homo 6-mer

^*a*^ The described systems include *A. thaliana* (5LNR, 7LB5) (28, 29), *B. subtilis* (2NV2) (27), *S. aureus* (8U7J) (30), *S. cerevisiae* (3FEM) (31), *P. berghei* (4ADS) (32), *T. maritima* (2ISS) (26), *T. thermophilus* (2ZBT) (33), *G. kaustophilus* (4WY0) (34), *G. stearothermophilus* (1ZNN) (35), *M. tuberculosis* (2ISS) (36), *M. jannaschii* (2YZR) (37), and *P. horikoshii* (4FIQ) (38).

### Structural analysis

**Fig 3:**
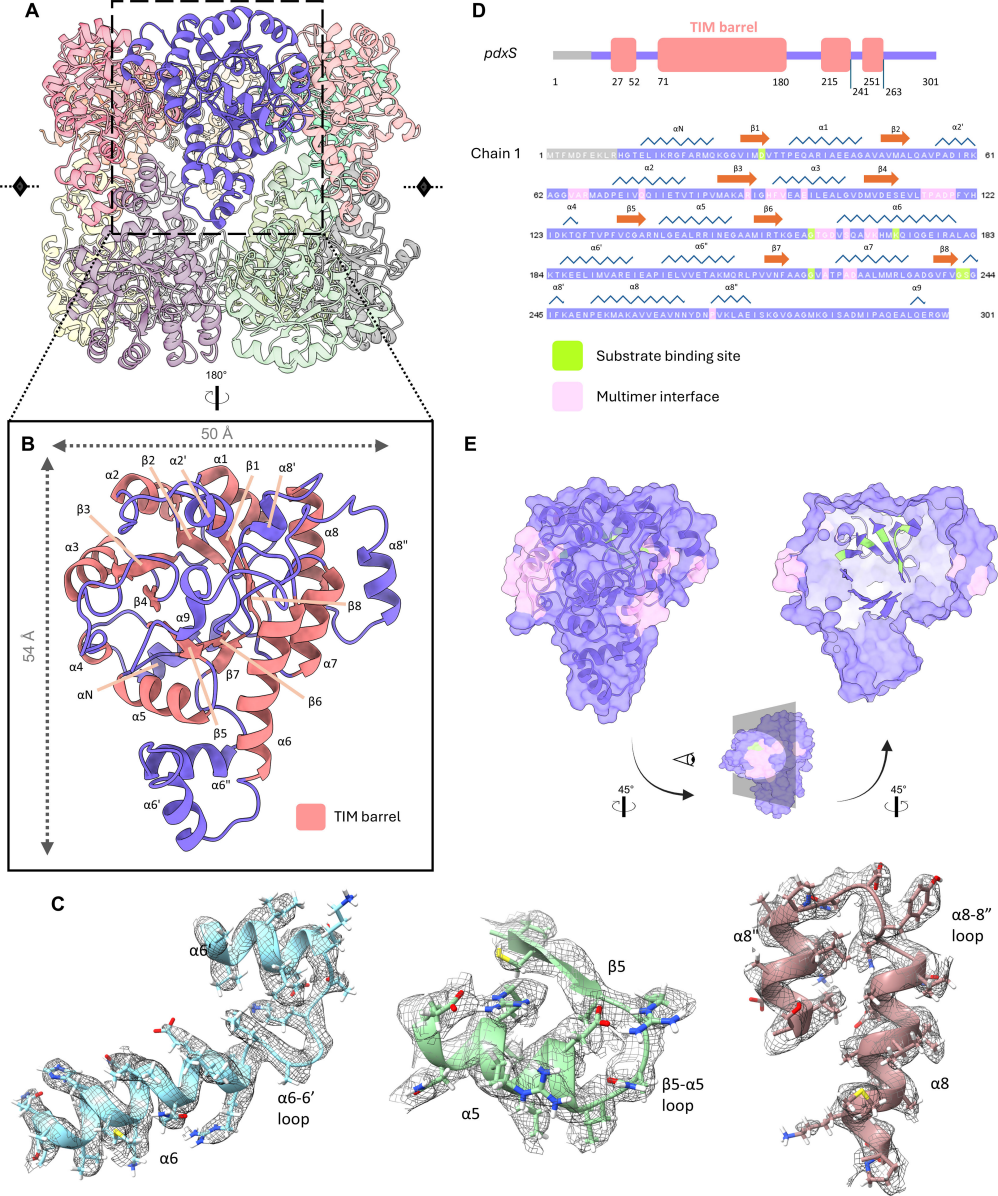
Monomer structure of PdxS. (**A**) Multimer structure of PdxS with one subunit highlighted. The monomer subunit is depicted as a ribbon representation with secondary structure elements labeled (**B**) and with selected sections docked into the cryo-EM density (represented as a mesh) to demonstrate sidechain fit (**C**). (**D**) Domain structure of the PdxS monomer and its complete primary sequence with secondary structure elements, binding site residues, and multimer interface interactions annotated according to UniProt entry (39). (**E**) Space-filling representation of the monomer model with multimer interfaces colored in pink and R5P binding site residues colored in green, along with a second cut-away view to reveal the beta barrel ribbon depiction within.

Like other homologous PLP synthase structures, the *M. acetivorans* PdxS dodecameric complex consists of two layers of homohexameric rings that stack cylindrically, with an outer diameter of 108 Å and an inner diameter of 42 Å (Fig. 2D). Each monomer adapts a triose phosphate isomerase (TIM) barrel fold that houses the active site for deoxyxylulose 5-phosphate-independent PLP synthesis from D-ribose 5-phosphate (R5P), D-glyceraldehyde 3-phosphate, and an ammonia moiety from glutamine. Ribulose 5-phosphate and dihydroxyacetone phosphate are also acceptable substrates, as PdxS homologs demonstrate triose and pentose isomerase activity in addition to PLP synthesis (as suggested by the domain architecture) (17). There is strong density in this binding pocket adjacent to residues annotated as binding sites for R5P, suggestive of captured particles undergoing native catalysis. The first 10 residues lack clear density for modeling, as was similarly observed with the *Arabidopsis thaliana* Pdx1.2/1.3 pseudoenzyme structures (28), indicating that this region is relatively flexible when unbound to its glutaminase, PdxT. The rest of the monomeric secondary structure elements are named by sequence, as depicted in Fig. 3B. There are in total 15 alpha helices and 8 beta sheets, with helices α1–8 (excluding prime and double-prime alpha helices) participating in the TIM-barrel fold with beta sheets β1–β8.

### Interactions between PdxS subunits

**Fig 4:**
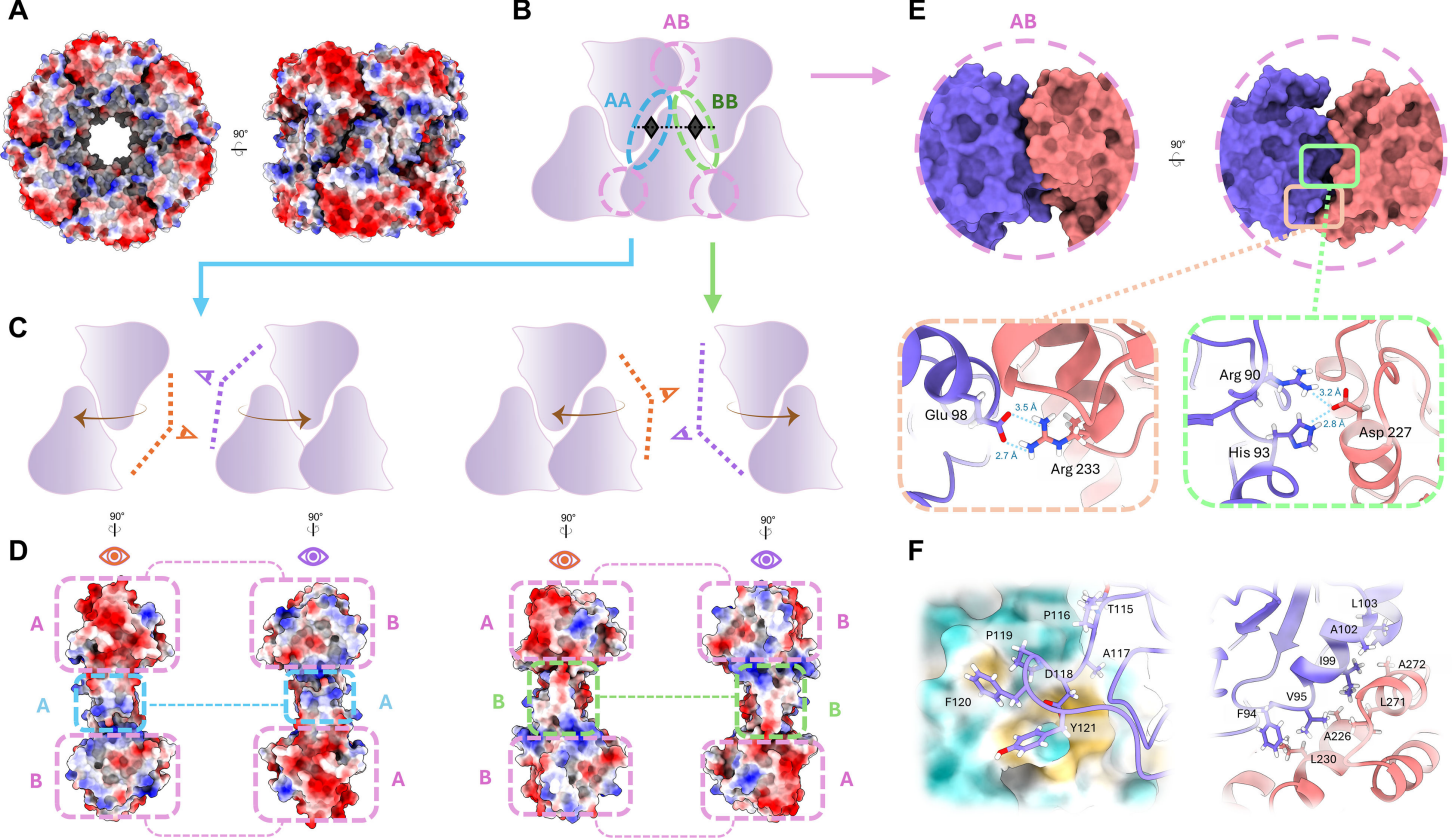
Charge and shape complementarity in oligomeric interfaces. (**A**) Top and side view of the space-filling representation of the PdxS dodecamer with surface electrostatics depicted in red (acidic) and blue (basic). (**B**) Cartoon schematic of intersubunit interactions defining each of three types of contacts, based on splitting the asymmetric monomer unit into two unique lateral faces, A and B. Each contact region is assigned a corresponding annotation color. (**C**) Illustration of views for each interface to be depicted in panel D. (**D**) Electrostatic surface coloring of complementary interfaces with each unique contact region annotated in its corresponding color as defined in (**B**). The AA and BB regions are self-contacts. Overall, acidic and basic patches along each surface are complementary. (**E**) Side and top views of the AB interface and select hydrogen-bonding partners contributing to its interface stabilization. (**F**) Representations of the hydrophobic loop (left panel) and hydrophobic helix-helix interactions (right panel) that stabilize lateral multimer binding. The left panel illustrates the B face of AB in a hydrophobicity surface coloring with yellow representing hydrophobic residues and the A face as a ribbon representation with projecting residues represented as sticks. The right panel shows the distribution of hydrophobic residues between face A’s α3 helix and face B’s α7 and α8′′ helices.

The atomic model of the PdxS complex shows high levels of surface and charge complementarity in its inter-subunit binding interfaces (Fig. 4). The monomer itself resembles an asymmetric, inverted triangle, demonstrating on either side a convex (A) or concave (B) facet posed for lateral binding. Monomer tips also interdigitate at the hexamer-hexamer interface, creating altogether three distinct types of lateral contacts that give rise to the dodecamer, here described as AB, AA, and BB (Fig. 4B). A network of six inter-subunit hydrogen bonds anneal the facets of interface AB together, while two such interactions comprise AA, and another four comprise BB (while each has 1 and 2 unique bonds, respectively, these are doubled due to the inherent twofold symmetry of the “self” interfaces). The residues contributing to the AB contacts are as follows. Two hydrogen bonds form between glutamate 98 of helix 3 and arginine 233 of helix 7 (Fig. 4E). Also posed on α7 is aspartate 227, which forms two hydrogen bonds; one with histidine 93 from loop β3 to α3, and arginine 90 on β3 (Fig. 4E). Arginine 67 from loop α2′ to α2 forms a backbone contact with methionine 282 of loop α8′′-α9. Lastly, aspartate 118 from loop β4 to α4 forms a hydrogen bond with lysine 172 of α6. The “self” interfaces (AA and BB) are dominated by electrostatic interactions between alpha helices 6 and 6′. Comprising the AA interface, the one unique sidechain bond pair is between arginine 194 (α6′) and glutamate 197 (α6′). For the contacts involved in BB, the two unique bonds are shared between arginine 179 (α6) and glutamate 188 (α6′).

The overall shape of the monomer is topologically complementary to itself, given that monomers within a hexamer self-associate via the concave-convex fit of the A and B interfaces. The alpha helices external to the (β/α)_8_ TIM barrel motif (αN, α2′, α6′, α6′′, α8′, α8′′, and α9) are also responsible for some morphological stabilization in addition to protecting the active site from solvent exposure (αN and α2′). These stabilizing contacts include a protrusion of the T115-Y121 loop into a hydrophobic patch along the crevice between the adjacent monomer’s α6 and α7 helices along the AB interface (Fig. 4F). Another such region is located at the same interface, anterior to the aforementioned loop; α7 and α8′′ share valine- and (iso)leucine-mediated contacts with the adjacent monomeric unit’s α3 helix (Fig. 4F).

In our maps, no instances of PdxT binding were observed, even after extensive 3D classification and examination under a low-density threshold. Furthermore, upon screening the higher molecular weight fractions of the glycerol density gradient, no PdxS-PdxT supracomplexes were detected as determined by 2D classification and examination of individual micrographs. In homologous proteins for which there are crystal structures, the αN helix of PdxS is instrumental to PdxT binding (40). The *M. acetivorans* αN helix displays several solvent-exposed hydrophobic residues (I16, G19, F20, M23), with regions of dense negative and positive charge above and below, respectively, which is highly suggestive of a putative binding interface for the glutaminase. That being said, given that this sample represents a composite of the most dominant states in our cellular lysate, our work may suggest a novel state wherein PdxT binds extremely transiently and, upon delivery of substrates, need not remain docked to the PdxS homo-dodecamer. This is supported by recent findings which demonstrate transient, sub-stoichiometric binding of Pdx1 and Pdx2 in *Staphylococcus aureus* PLP synthase by SAXS (30).

### Active site chemistry and evolutionary conservation

**Fig 5:**
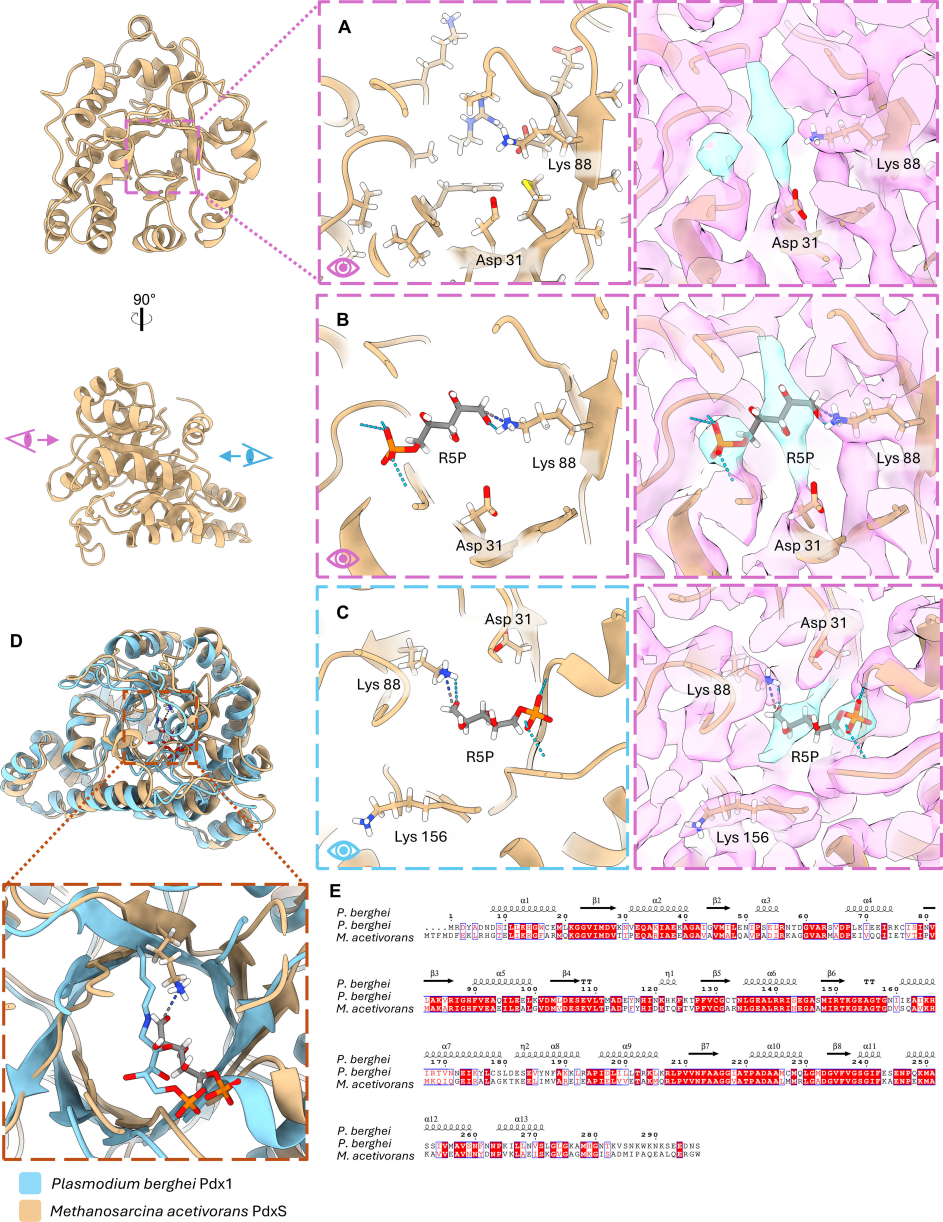
Active site densities and potential ligand modeling. (**A**) Ribbon model and corresponding cryo-EM density of PdxS in its active site. Nearby active site residues are represented as stick models. The elongated density and its neighboring spherical density colored in blue are not occupied by any nearby side chains. (**B and C**) Possible R5P configuration in active site density, with the phosphate group docked into the spherical density. A potential Schiff base is represented with K88 as a dashed dark blue and gray line. Hydrogen bonds between the phosphate group and nearby backbone are depicted with a dashed light blue line. (**C**) Alternative view of the docked R5P, revealing the strong density for the second active sight lysine (K156) in its extra-active site conformation. (**D and E**) Comparison of *Plasmodium berghei* and *M. acetivorans* Pdx structure (**D**) and primary sequence (**E**). The crystal structure for *P. berghei* bound with R5P is aligned with our structure, including our hypothesized R5P placement in the density.

On the interior of the TIM barrel, we observe an elongated density adjacent to residues annotated as substrate-binding sites (Fig. 5A). The PLP synthase active site chemistry as currently understood is rather complex, involving ring openings, closings, and isomerizations but generally proceeds with a linearized R5P being stabilized via formation of a Schiff base of its C1 with an active site lysine residue (40). A secondary lysine residue likewise bonds with the C5 of R5P and is eventually responsible for swinging the intermediate to a second phosphate-binding site exterior to the TIM barrel (29). Based on comparison with previous crystal structures where PdxS was co-crystallized with R5P and various intermediate states (29), the density is most likely to belong to either bound R5P or an R5P Schiff-base intermediate. Docking an R5P moiety with the molecule oriented such that the phosphate group is positioned in the bulbous density nestled between β6 and loops 156–164 then positions the oxygen 1 of R5P next to lysine 88, which is where the structurally equivalent lysine in other models (e.g., *Plasmodium berghei*) forms a Schiff-base intermediate with R5P (Fig. 5B through E). This also positions R5P to engage in a hydrogen bond with aspartate 31. One caveat to this assignment is that the oblong density adjacent to the putative phosphate density does, however, run in a perpendicular direction to the conserved R5P position. Repositioning the R5P to align with this perpendicular density could suggest the engagement of the second active site lysine (K156) in the formation of a second Schiff base with C5, but the density in our map for K156 is very strongly oriented in its alternate conformation facing away from the TIM barrel active site (Fig. 5C). It is clear, therefore, that multiple substrate states have likely been captured in this averaged density map.

**Fig 6:**
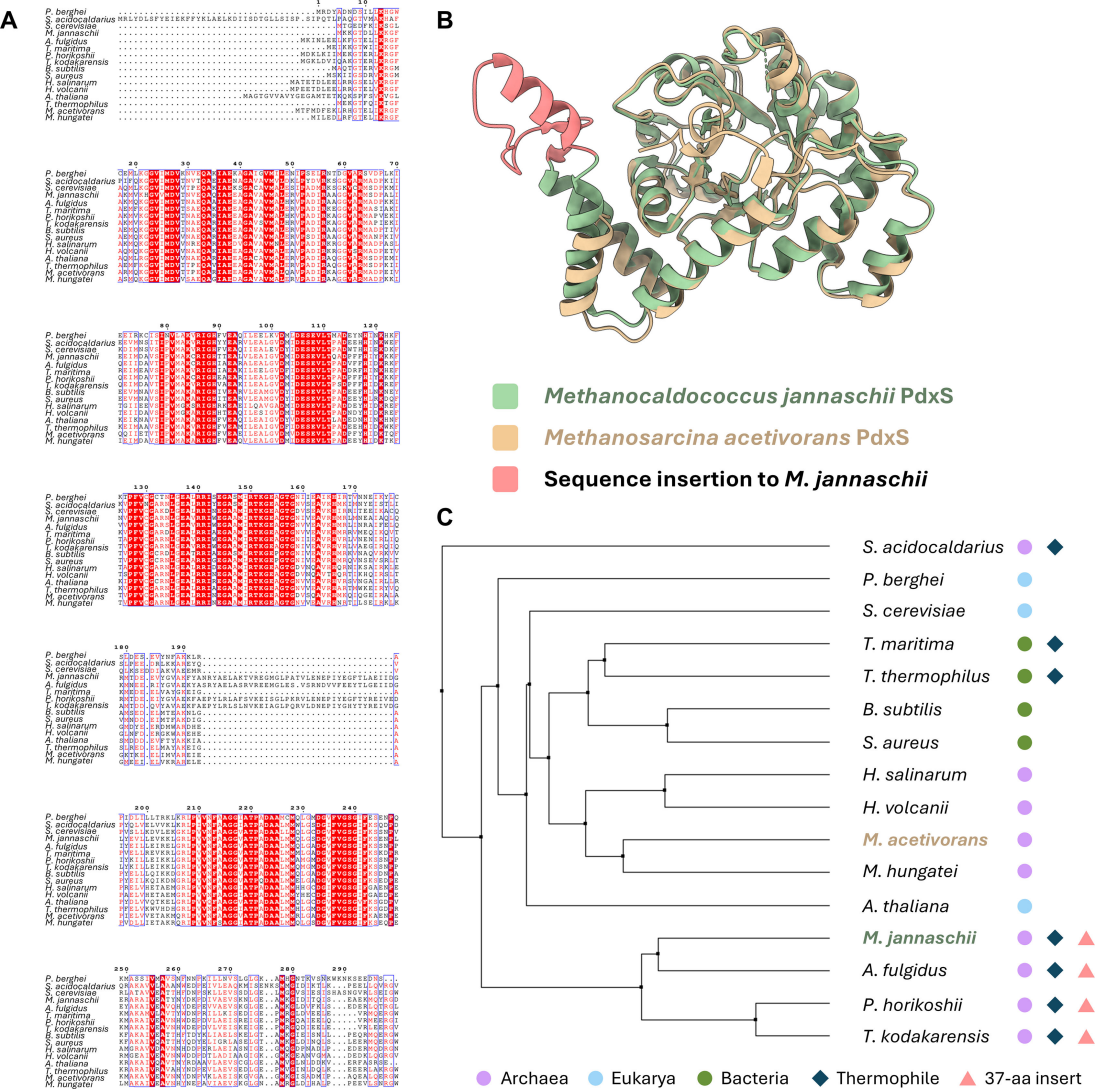
Sequence insertions in previously deposited archaeal PLP synthase subunits and other thermophilic archaeal species. (**A**) Sequence alignment between select species for which Pdx1/S structures have been deposited and additional thermophilic and mesophilic archaea. *P. horikoshii* and *M. jannaschii* both contain 37-aa inserts that are lacking in all other deposited Pdx1/S structures. (**B**) Structure alignment of the *M. jannaschii* and *M. acetivorans* PdxS monomers, with the sequence insertion in *M. jannaschii* highlighted in coral. (**C**) Average distance tree based on the ClustalW sequence alignment of primary sequences listed in (**A**). Domain, thermophilicity, and presence of a sequence insertion are denoted by corresponding symbols. Note that *Sulfolobus acidocaldarius* uniquely contains an N-terminal insertion instead, of also 37 amino acids.

Upon performing a phylogenetic analysis of the PLP synthase sequences from other biological systems for which crystal structures have been deposited, it is apparent that the *M. acetivorans* PdxS shares more sequence and structural similarity with the other deposited bacterial and eukaryotic PLP synthases than with the two archaeal systems so far determined (*Pyrococcus horikoshii* and *Methanococcus jannaschii*, Fig. 6) (37, 38). This is due to a 37-aa insertion that appears in various classes across several archaeal phyla, including Euryarchaeota (a kingdom shared with *M. acetivorans*), though notably this mainly appears within thermophilic genera (Fig. 6). The insertion gives rise to an extra alpha helix and beta sheets between equivalent helices to *M. acetivorans* α6′ and α6′′.

## DISCUSSION

In this study, we have determined the first native structure of the PdxS component of PLP synthase from an archaeon by cryo-EM and the *cryoID* approach. We demonstrate that the high degree of structural and sequence conservation witnessed in other crystal structures across bacteria and eukaryotes is also shared in this archaeal lineage. Intriguingly, our model shows greater divergence from the sole deposited archaeal PLP synthase structures at current than all other taxa for which the synthase structure is known. Given that those two species are extremophiles, the sequence insertion may have arisen due to their high-temperature conditions. Indeed, the additional helix and loops provide further electrostatic and hydrophobic interactions between subunits within hexamers, potentially further stabilizing the oligomerized state in the face of immense heat from these thermophilic organisms’ environment (38). This is further supported by sequence alignments with other thermophilic and mesophilic archaea, which reveal that these sequence insertions are confined to PdxS sequences from archaeal thermophiles (Fig. 6A). Indeed, the top 100 hits from an NCBI BLAST search for this insertion contain only organisms from the extreme thermophilic Euryarchaeota lineage. Interestingly, the extreme thermophilic Proteoarchaeota (*Sulfolobus acidocaldarius*), the mesophilic Euryarchaeota, and bacterial extreme thermophiles lack the 37-aa insert noted in *P. horikoshii* and *M. jannaschii*, suggesting a relatively ancient evolutionary divergence from the Euryarchaeota thermophiles as well as indicating the existence of two structurally distinct classes of thermophilic PLP synthases (Fig. 6C).

Notably, density corresponding to bound PdxT was absent from our refined structure. Even after focused classification, evidence for any significant population of PdxT could not be observed. Prior crystal structures demonstrate 24-mer complexes but only under conditions of extremely high protein concentration, which are intrinsically required for crystallization. This suggests that natively, PdxT may bind the homo-dodecameric complex very weakly. It has been recently shown that PdxS can even function with direct ammonia binding, though ammonia delivery via the glutaminase promotes the efficiency of the synthase (30). Still, perhaps this indicates that the intracellular occupancy or expression of PdxT is often low. The aforementioned study also finds that in the *S. aureus* PLP synthase, the majority of particles form sub-stoichiometric complexes, with the number of homo-dodecamers outnumbering 24-mers, which supports the notion that the PdxS-PdxT interaction is generally unstable.

Our present work also demonstrates the promise of leveraging the *cryoID* workflow for native proteins harvested directly from cellular lysate. With only crude size-based fractionation, current single-particle analysis capabilities were successful in selecting, reconstructing, and identifying a native protein. Furthermore, we observe in our density some evidence of active site occupancy. Because of the multiple orientations of R5P or R5P intermediates this density accommodates, it is likely that we captured an amalgamation of many catalytic states. With further imaging and a greater particle set, these states can be classified and separately reconstructed. As such, this approach is particularly promising for studying native interactions involved in catalytic cycles of enzymes or for those systems which lack developed or established molecular biology techniques for extensive gene editing, endogenous protein purification or require unique archaeal cofactors. Structural characterization of biomolecules is crucial to (i) the development of targeted therapies in disease-causing systems and (ii) the pursuit of evolutionary descriptions of function. Therefore, it is essential to leverage the gross information retrieval capabilities of bottom-up cryo-EM toward understudied organisms, such as those in archaeal taxa.

## MATERIALS AND METHODS

### Sample preparation

*M. acetivorans* C2A (DSM 2834) was cultivated in 100 mL serum bottles with an N_2_:CO_2_ (80:20) headspace and 50 mL medium as previously described (41). Methanol was used as the sole carbon and energy supply (50 mM). Cells were harvested at OD ~1 by centrifugation at 10,000 × *g* for 10 min at 5°C in an IEC tabletop centrifuge. Cells were resuspended in chilled lysis buffer (137 mM NaCl, 2.7 mM KCl, 10 mM Na_2_HPO_4_, 1.8 mM KH_2_PO_4_, 5 mM MgCl, 2 mM dithiothreitol, and protease inhibitor cocktail) and lysed via 1.5–150 Watt adjustable sonication at 20–25 kHz (40% power using ten 2 s pulses at 4°C). Cell lysate was incubated with benzonase for 10 min before centrifugation (12,000 × *g* for 6 min at 4°C) to clarify nucleic acid and insoluble cellular debris. The supernatant was decanted, applied to a 10%–30% glycerol density gradient, and centrifuged at 110,000 × *g* for 20 hours at 4°C. Furthermore, 500 µL gradient fractions were evaluated by SDS-PAGE and negative stain electron microscopy, and selected fractions containing particles 8–12 nm in diameter were pooled together for subsequent grid preparation.

### Electron microscopy of stained and vitrified samples

For negative stain EM screening, 3 µL aliquots were applied to glow-discharged formvar/carbon-coated grids (300 mesh, Ted Pella) and incubated for 1 min, before negative staining with 2% uranyl acetate. Samples were screened on an FEI Tecnai F20 electron microscope operated at 200 keV, and images were recorded on a TIETZ F415MP 16-megapixel CCD camera at a nominal magnification of 60,000×.

Prior to freezing, n-dodecyl-beta-maltoside was added to the sample to a final concentration of 0.0043% (one half the CMC) to alleviate protein denaturation arising from aggregation at the air-water interface. Subsequently, 3 µL aliquots were applied to glow-discharged holey carbon grids (Quantifoil R1.2/1.3 300 mesh, Ted Pella) and incubated for 10 s before automated blotting and flash freezing in liquid ethane with a Vitrobot Mark IV vitrification system (Thermo Fisher Scientific). Various freezing conditions—including chamber temperature, humidity, blotting time, blotting force, and drain time after blotting—were screened on the aforementioned instrument used during negative stain evaluation. Optimal conditions were obtained with a 90-s glow discharge, a chamber temperature of 4°C and 100% humidity, 5-s blotting time, blot force of 0, and 0-s drain time. Optimized cryo-EM grids were stored in liquid nitrogen until cryo-EM data collection.

Imaging for downstream processing was performed on a Titan Krios 300 kV electron microscope (Thermo Fisher Scientific) equipped with a Gatan Imaging Filter (GIF) Quantum LS and a Gatan K3 direct electron detector. A total of 14,890 movies were recorded with SerialEM in super-resolution mode at a nominal magnification of 81,000×, yielding a calibrated pixel size of 0.55 Å/pixel at the specimen level (42). The GIF slit width was set to 20 eV. Each movie contained 44 frames with an exposure time of 2.2 s per frame, giving a total estimated electron dose of 50 e^−^/ Å^2^.

### Structure determination

Movie frames were aligned with patch motion correction in the cryoSPARC v4 suite, after which the calibrated pixel size was 1.1 Å/pixel (24). Defocus values were determined with the cryoSPARC patch CTF estimation job. Autonomous, reference-free particle picking was first performed with the Blob Picker tool, specifically selecting for particles in the size range of 80–120 Å diameter. Initially 14,627,499 particles were picked from 14,890 micrographs and boxed out by 300 × 300 pixels. After several iterations of 2D classification to remove junk classes, three distinct classes demonstrating alignment to 6 Å resolution emerged. These classes were used as templates to repick the data set, which after iterative 2D classification yielded 17 well-aligned classes of 614,949 particles. This particle subset was subsequently used to train the deep learning-based particle picker Topaz, after which the entire data set was again repicked with the trained model, yielding 3,724,083 particles (25). Another round of iterative 2D classification gave rise to many well-aligned, distinct 2D classes. Of these, several classes appeared to represent a 100 Å D6-symmetric structure. These were used for the training of a separate Topaz model, and the data set was picked a third time with a model trained on just these views. From the resultant 2D classes, a total of 99,971 particles were selected and subjected to *ab initio* reconstruction in cryoSPARC.

This initial model was used as a reference for nonuniform refinement, and with D6 symmetry enforced, the map was reconstructed to 3.59 Å. After 3D classification and iterative CTF refinement and 3D refinement, the final resolution was 3.38 Å based on the gold-standard FSC 0.143 criterion. B-factor, local resolution, and FSC curves were all calculated in cryoSPARC.

### Structure identification with cryoID

After feeding *cryoID* our sharpened map, the program generated two query sequences built into two helical domains. The model was extended on both termini as permitted by the density, resulting in the following query sequences: (i) GGKYLKLPLGLGKGLGGKGLL and (ii) LPGKLGLGG. The top ranked output was UniProt Q8TQH6, or PdxS, which was further validated by docking the Alphafold3-predicted structure into our density.

### Atomic modeling, model refinement, and graphics visualization

Modeling was performed by docking the Alphafold3-predicted structure of the PdxS monomer into the cryo-EM density and confirming that the output identification was rational. The model was then iteratively real space refined in the ISOLDE package of UCSF ChimeraX and validated in PHENIX, until outlier scores became negligible (43–45). A last round of real space refinement was performed in PHENIX and evaluated using the PDB validation server (46). Visualization of all maps and models was performed with UCSF ChimeraX. All sequence alignments were performed with ClustalW and visualized with ESPript 3 (47, 48). The average distance tree was constructed with JalView (49).

## Data Availability

The PdxS cryo-EM map has been deposited in the Electron Microscopy Data Bank under accession number EMD-47202. The coordinates of the PdxS model have been deposited in the Protein Data Bank under accession number 9DVF. This paper does not report original code. Any additional information required to reanalyze the data reported in this paper is available from the lead contact upon request.
